# Repurposing BCL-2 and Jak 1/2 inhibitors: Cure and treatment of HIV-1 and other viral infections

**DOI:** 10.3389/fimmu.2022.1033672

**Published:** 2022-12-09

**Authors:** Monica D. Reece, Colin Song, Sarah C. Hancock, Susan Pereira Ribeiro, Deanna A. Kulpa, Christina Gavegnano

**Affiliations:** ^1^Department of Pathology and Laboratory Medicine, School of Medicine, Emory University, Atlanta, GA, United States; ^2^Department of Chemistry, College of Arts and Sciences, Emory University, Atlanta, GA, United States; ^3^Department of Biology, College of Arts and Sciences, Emory University, Atlanta, GA, United States; ^4^Department of Pharmacology and Chemical Biology, School of Medicine, Emory University, Atlanta, GA, United States; ^5^Center for the Study of Human Health, College of Arts and Sciences, Emory University, Atlanta, GA, United States; ^6^Department of Pathology and Laboratory Medicine, Atlanta Veterans Affairs Medical Center, Decatur, GA, United States; ^7^Center for Bioethics, Harvard Medical School, Boston, MA, United States

**Keywords:** HIV-1, Jak Stat, cancer, Bcl-2, inflammation, HIV reservoir, immunosenecence

## Abstract

B cell lymphoma 2 (BCL-2) family proteins are involved in the mitochondrial apoptotic pathway and are key modulators of cellular lifespan, which is dysregulated during human immunodeficiency virus type 1 (HIV-1) and other viral infections, thereby increasing the lifespan of cells harboring virus, including the latent HIV-1 reservoir. Long-lived cells harboring integrated HIV-1 DNA is a major barrier to eradication. Strategies reducing the lifespan of reservoir cells could significantly impact the field of cure research, while also providing insight into immunomodulatory strategies that can crosstalk to other viral infections. Venetoclax is a first-in-class orally bioavailable BCL-2 homology 3 (BH3) mimetic that recently received Food and Drug Administration (FDA) approval for treatment in myeloid and lymphocytic leukemia. Venetoclax has been recently investigated in HIV-1 and demonstrated anti-HIV-1 effects including a reduction in reservoir size. Another immunomodulatory strategy towards reduction in the lifespan of the reservoir is Jak 1/2 inhibition. The Jak STAT pathway has been implicated in BCL-2 and interleukin 10 (IL-10) expression, leading to a downstream effect of cellular senescence. Ruxolitinib and baricitinib are FDA-approved, orally bioavailable Jak 1/2 inhibitors that have been shown to indirectly decay the HIV-1 latent reservoir, and down-regulate markers of HIV-1 persistence, immune dysregulation and reservoir lifespan *in vitro* and *ex vivo*. Ruxolitinib recently demonstrated a significant decrease in BCL-2 expression in a human study of virally suppressed people living with HIV (PWH), and baricitinib recently received emergency use approval for the indication of coronavirus disease 2019 (COVID-19), underscoring their safety and efficacy in the viral infection setting. BCL-2 and Jak 1/2 inhibitors could be repurposed as immunomodulators for not only HIV-1 and COVID-19, but other viruses that upregulate BCL-2 anti-apoptotic proteins. This review examines potential routes for BCL-2 and Jak 1/2 inhibitors as immunomodulators for treatment and cure of HIV-1 and other viral infections.

## Introduction

B cell lymphoma 2 (BCL-2) family proteins fall into three categories: anti-apoptotic proteins (BCL-2, BCL-X_L_, BCL-W, MCL-1, BFL-1/A1), pro-apoptotic pore-formers (BAX, BAK, BOK), and pro-apoptotic BH3-only proteins (BAD, BID, BIK, BIM, BMF, HRK, NOXA, PUMA) (1, 2). BCL-2 proteins have a large role in regulating apoptosis, and dysregulation can lead to oncogenesis and senescence. Several intracellular pathogens and/or cancers have developed mechanisms to avoid cellular death to favor its own survival. BCL-2 is a key modulator of cellular lifespan and has been shown to be modulated by pathogen/host interactions and by certain cytokines downstream of viral infection and/or tumor microenvironment. Thus, the development of inhibitors of BCL-2 proteins has been of great interest for the virus infection and cancer fields. Several BCL-2 inhibitor trials have taken place and shown efficacy in inducing apoptosis resulting in better disease outcomes. In this review we will highlight the induction of BCL-2 by different diseases (viral and cancerous), its association with immune senescence and exhaustion, and Food and Drug Administration (FDA) approved drugs that interfere with survival pathways leading to better disease outcomes.

## BCL-2 induction

### Jak STAT cascade for BCL-2 expression

**Figure 1:**
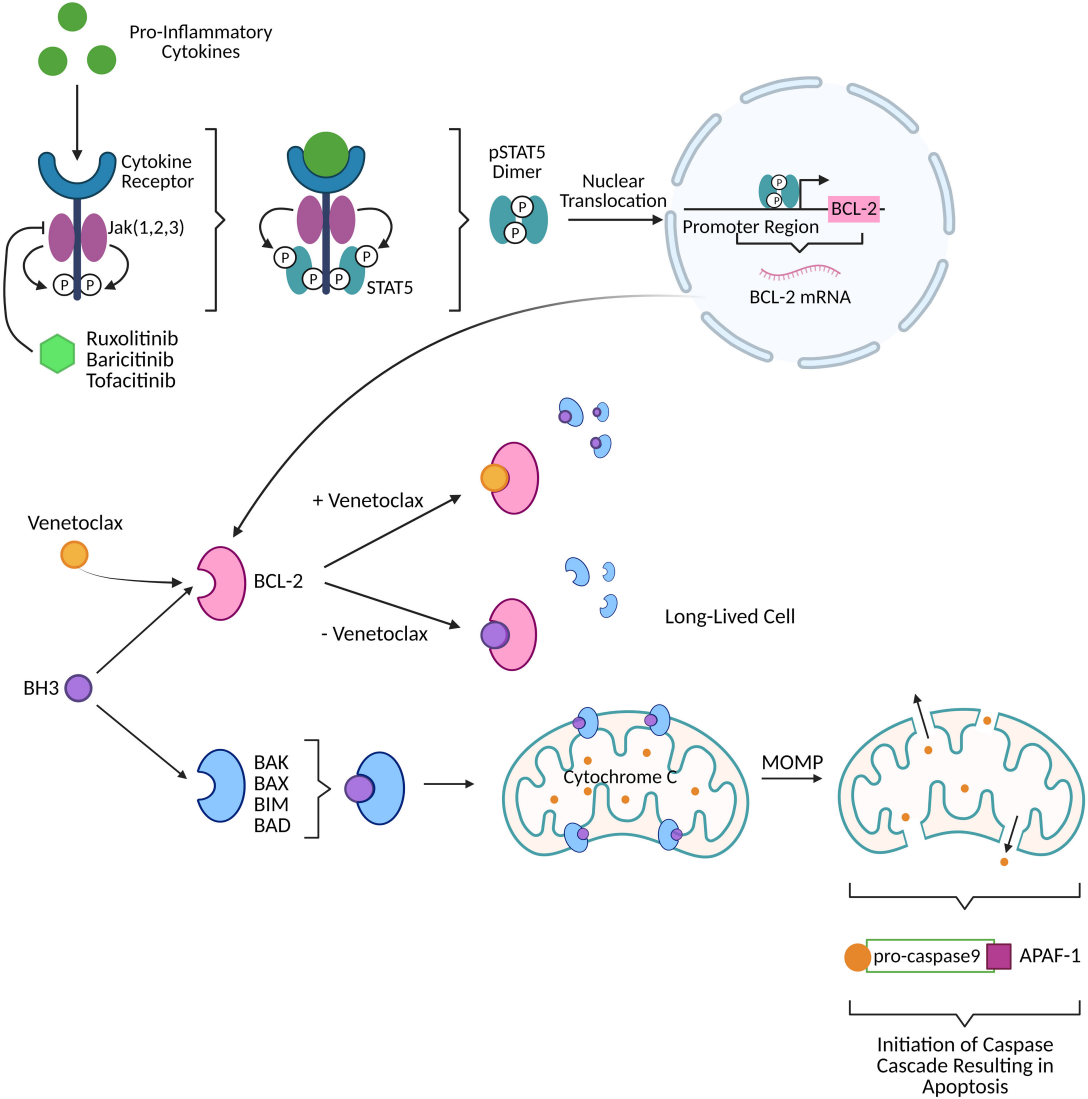
Activation of the Jak STAT pathway by pro-inflammatory cytokines (like those produced in viral infection) produces dimerized pSTAT5 that directly upregulates BCL-2, a pro-survival factor, resulting in long-lived cells harboring HIV-1 DNA. FDA approved Jak inhibitors (ruxolitinib, baricitinib, tofacitinib) can block the BCL-2 cascade upstream. Venetoclax, FDA approved BCL-2 selective BCL-2 homology 3 (BH3) mimetic, prevents BCL-2 sequestration of BH3. Free BH3 is then able to interact with pro-apoptotic proteins (BAK, BAX, BIM, BAD) to perform mitochondrial outer membrane permeabilization (MOMP) thereby releasing cytochrome C. Cytochrome C interacts with pro-caspase9/apoptotic protease activating factor 1 (APAF-1) to initiate a caspase cascade that culminates in apoptotic cell death. Created with BioRender.

The Janus kinase and signal transducer and activator of transcription (Jak STAT) pathway is an upstream regulator of BCL-2 expression. When proinflammatory cytokines engage their cytokine receptor, Janus kinases (Jak) 1, 2, and 3 phosphorylate the receptor which recruits STAT type 5 (STAT5). STAT5 is subsequently phosphorylated (pSTAT5) by Jak 1, 2, and 3 and dimerizes. Dimerized pSTAT5 undergoes nuclear translocation and binds to the promoter region of the BCL-2 gene to enhance transcription (**Figure 1**) (3–6). Elevated levels of BCL-2, pro-survival factor, are a marker of pathogenesis for many diseases including cancer and human immunodeficiency virus type 1 (HIV-1). Targeting the Jak STAT pathway has thus far proven to decrease BCL-2 expression while also decaying the HIV-1 reservoir and reversing HIV-1-associated neurocognitive dysfunction caused by long-term basal inflammation (3, 7–9). Broadly, pSTAT5 expression has been linked to increased integrated HIV-1 DNA levels *in vivo*, *in vitro*, and *ex vivo*, deregulated homeostatic proliferation, immune activation, reservoir size *in vivo*, and BCL-2 expression *in vitro* and *in vivo* (3, 7). Additionally, STAT5 phosphorylation is involved in homeostatic proliferation (3) and has been reported to have binding sites in the HIV-1 long terminal repeat (LTR) (10).

## Role of BCL-2 expression

### Cancers

BCL-2 has been extensively studied in cancer, with the original BCL-2 inhibitor intended as a cancer treatment. Within the BCL-2 family of proteins, there are both pro- and anti-apoptotic proteins. The anti-apoptotic proteins contribute to cellular senescence and tumor survival. Senescence is a cellular response to physiological and oncogenic stress that results in the loss of a cell’s ability to divide and grow (11). Anti-apoptotic BCL-2 family proteins are upregulated in senescent cells (11). Two major apoptotic pathways that BCL-2 proteins play a critical role in are the intrinsic cell-death pathway, also called the mitochondrial apoptotic pathway, and the extrinsic cell-death pathway involving the FAS and TRAIL receptors that initiate a caspase-8-mediated cascade (12). Within the intrinsic cell-death pathway, anti-apoptotic proteins such as BCL-2, BCL-X_L_, and MCL-1 possess a C-terminal transmembrane domain anchored in the mitochondrial outer membrane. High expression of BCL-2 or BCL-X_L_ is associated with aggressive malignancies and resistance to chemotherapy (12). BAK and BAX are pro-apoptotic proteins that are blocked by BCL-2, BCL-X_L_, and MCL-1. The BH3 peptide activates BAK and BAX but is sequestered by BCL-2, BCL-X_L_, and MCL-1 through binding to a hydrophobic pocket in the protein. When BAK and BAX are successfully activated, they oligomerize in the mitochondrial outer membrane causing mitochondrial outer membrane permeabilization (MOMP). This membrane damage not only causes cellular necrosis but also allows for release of proteins from the mitochondria that contribute to apoptosis. The proteins include caspase activating proteins like cytochrome C, caspase inhibitor neutralizers like SMAC and OMI/Htra2, and intranuclear genome degradation proteins like apoptosis-inducing factor (AIF) (13). Cytochrome C combines with both apoptosis protease-activating factor 1 (APAF-1) and inactive procaspase-9 to form an apoptosome. This formation activates caspase-9 which initiates a caspase cascade involving caspase-3, caspase-6, and caspase-7 (**Figure 1**) (12). BCL-2 anti-apoptotic proteins’ ability to subvert apoptosis can result in a senescent cellular phenotype where the cell is not actively proliferating but in stasis. Cytokines from the tumor microenvironment such as transforming growth factor beta (TGF-β) and interleukin 10 (IL-10) are known cytokines to promote tumor survival but also to induce immunosenescence by upregulating BCL-2 expression in tumor and immune cells as well as the expression of co-inhibitory receptors (preliminary data from our group DOI: 10.1101/2020.12.15.422949, 10.1101/2021.02.26.432955). While immunosenescence primarily refers to the changes in the immune system associated with age and impacts mainly T lymphocytes; chronic diseases such as cancers and certain infectious diseases (cytomegalovirus (CMV), HIV-1) are also associated with immune dysfunction.

### BCL-2 and HIV-1 reservoir persistence

In 2020 in the United States, around 30,69200 people were newly diagnosed with human immunodeficiency virus type 1 (HIV-1), joining a total population of 1.07 million people with HIV (PWH) [CDC 2020 HIV surveillance report] (14). Globally, the UNAIDS report estimates 38.4 million PWH with 1.5 million new infections in 2021 [UNAIDS 2022] (15). Antiretroviral therapy (ART) is the common treatment regimen prescribed to PWH, but several social, geographic, and monetary blockades impact access and adherence to treatment resulting in 66-85% of PWH accessing treatment globally in 2021 [UNAIDS 2022] (15). In 2018, the cost of initial ART in the United States ranged from $36-48,000 annually, reducing accessibility and adherence to ART (16). ART can suppress HIV-1 replication to undetectable levels but is not able to eliminate it entirely due to the virus’s ability to form a population of HIV-1-infected cells with a transcriptionally silent LTR, commonly referred to as the HIV-1 latent reservoir. Despite the effectiveness of ART, life-long adherence to treatment with sustained viral suppression is complicated by the development of drug resistance, intolerance, and interactions, the earlier and more severe onset of diseases associated with aging, and social and psychological stigmas. If ART is interrupted, the HIV-1 reservoir contributes to the rapid rebound of plasma viremia in all but rare HIV-1 controllers (16, 17). Viral rebound not only increases viral load, thus increasing probability of spreading the virus to others, but also contributes to further immune system degradation and eventual progression into acquired immunodeficiency syndrome (AIDS). Eliminating or completely repressing the HIV-1 reservoir is critical for a true or functional cure, respectively. One approach to cure is the shock and kill method, which involves “shocking” the latent cells out of latency to become transcriptionally active which not only results in viral cytopathic effects but also allows infected cells to be killed by CD8+ T cells. Although this strategy continues to be investigated, there has been no efficacy demonstrated in humans to date. A functional cure refers to viral remission, a similar concept to cancer treatment, in which the viral reservoir is suppressed permanently and monitored for rebound. Jak inhibitors and BH3 mimetics reveal an alternate route in which we may be able to target the HIV reservoir while maintaining viral suppression and targeting downstream effects on the system (chronic inflammation, senescence, etc.), neither reactivating virus nor enforcing remission, but targeting dormant reservoir for decay.

Several mechanisms are in place leading to long term HIV-1 reservoir persistence. We have reported that IL-10 signaling leads to the upregulation of pro-survival pathways resulting in higher HIV-1 reservoir (preliminary data from our group DOI: 10.1101/2021.02.26.432955). *In vitro* blockade of IL-10 signaling led to BCL-2 downregulation and HIV-1 reservoir decay. Importantly, IL-10 also led to the upregulation of co-inhibitory receptors such as PD-1, supporting HIV-1 latency, but also CD8 T cells senescence and exhaustion.

**Figure 2:**
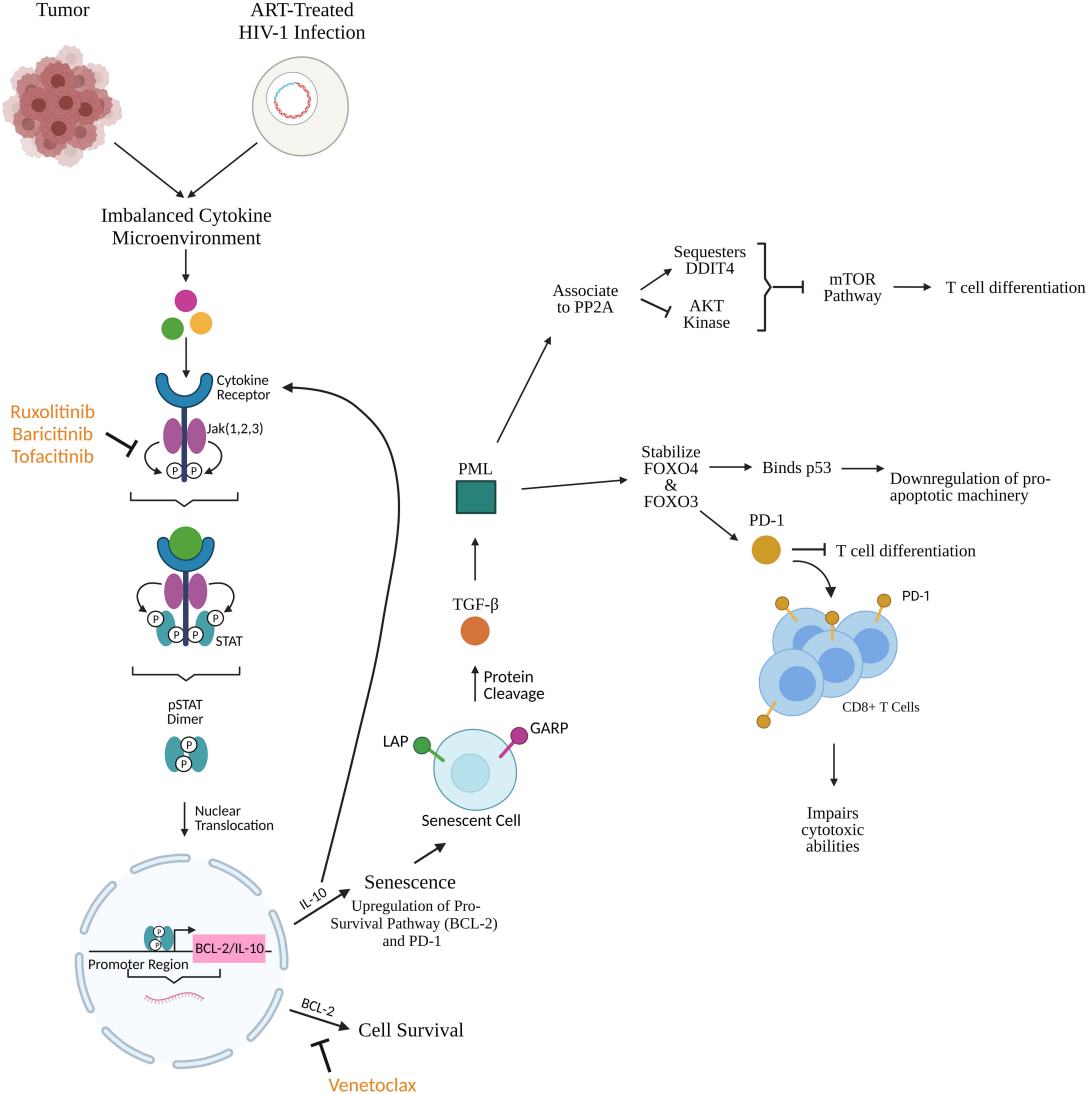
Cytokines produced subsequently from tumors and HIV-1-infected cells initiate the Jak STAT pathway leading to upregulation of pro-survival and pro-senescence factors like BCL-2 and IL-10. Jak inhibitors (ruxolitinib, baricitinib, tofacitinib) prevent upregulation of these factors upstream while BCL-2 homology 3 (BH3) mimetics like venetoclax prevent BCL-2 function downstream, as shown in **Figure 1**. IL-10 promotes cellular senescence through upregulation of BCL-2 and programmed death protein 1 (PD-1). Senescent cells express latency-associated peptide (LAP) and glycoprotein A repetitions predominant (GARP) which undergo protein cleavage and release bound TGF-β. TGF-β upregulates promyelocytic leukemia protein (PML) which associates to protein phosphatase 2A (PP2A) thereby sequestering DNA damage inducible transcript 4 (DDIT4) and inhibiting Ak strain transforming (AKT) kinase, ultimately resulting in inhibition of the mammalian target of rapamycin (mTOR) pathway which is responsible for T cell differentiation. In parallel, PML stabilizes forkhead box O4 (FOXO4) and O3 (FOXO3). FOXO4 binds p53 to downregulate pro-apoptotic machinery. FOXO3 upregulates PD-1 expression on CD8+ T cells which blocks T cell differentiation and impairs cytotoxic ability. This creates a phenotype of long-lived senescent cells and T cells with impaired cytotoxic/killing potential further maintaining the HIV-1 reservoir. Created with BioRender.

STAT type 3 (STAT3) produced *via* the Jak STAT pathway translocates to the nucleus and binds to the IL-10 promoter to induce expression. IL-10 causes a senescent phenotype by impeding cytokine production, prevention MHC upregulation, and inhibiting tyrosine phosphorylation in T cells which leads to a decrease in proliferation, differentiation, and IFN-γ and IL-2 production (preliminary data from our group DOI: 10.1101/2021.02.26.432955). Senescent cells in HIV-1 infection are characterized by expression of biomarkers like latency-associate peptide (LAP) and glycoprotein A repetitions predominant (GARP) (preliminary data from our group DOI: 10.1101/2020.12.15.422949), the inactive form of TGF- β in the cell surface. TGF-β is a known latency inducer in HIV-1 (18). GARP/LAP cleavage increases active plasma levels of TFG-β which initiates a signal transduction cascade that leads to upregulation of promyelocytic leukemia protein (PML). PML can either establish cellular quiescence by stabilizing forkhead box O3 (FOXO3) and O4 (FOXO4), or it can, in response to cellular stress, associate to protein phosphatase 2A (PP2A). This association inactivates AKT kinase and sequesters DNA damage inducible transcript 4 (DDIT4) which inhibits the mammalian target of rapamycin (mTOR) pathway which triggers effector differentiation and glycolysis. FOXO4 binds p53 tumor suppressor and downregulates pro-apoptotic machinery (preliminary data from our group DOI: 10.1101/2020.12.15.422949). IL-10 induces activators of STAT3 and promotes STAT3 translocation, ultimately upregulating expression of downstream proteins including p53 (19). FOXO3 upregulates PD-1, a T cell response regulator and marker of disease progression, blocking T cell differentiation (preliminary data from our group DOI: 10.1101/2020.12.15.422949) (20). PD-1 interactions with ligands PD-L1 and PD-L2 tolerize T cells to antigens to defuse T cell effector functions (20–22). Ultimately, downregulation of pro-apoptotic machinery would increase availability of anti-apoptotic machinery and further contribute to HIV-1 reservoir persistence (**Figure 2**). α-PD-1 monoclonal antibodies reverse T cell exhaustion in cancer patients and restore anti-tumor potential of T cells (20) Further upstream, blocking IL-10 inhibits upregulation of PD-L1 and α-IL-10 decreases frequency of PD1+ cells (preliminary data from our group DOI: 10.1101/2021.02.26.432955) (23). We have shown that TFG-β signaling is associated to heightened inducible HIV-1 reservoir and also to lack of central memory T cell differentiation, associated with increased expression of PD-1 (preliminary data from our group DOI: 10.1101/2020.12.15.422949), confirming the importance of this pathway in HIV-1 persistence and immune senescence. As proposed previously (preliminary data from our group DOI: 10.1101/2020.12.15.422949), combining PD-1 and TGF-β focused therapies with senolytics will improve the therapeutic impact on senescent cells, including CD8+ T cell cytolytic functions, in HIV-1 and cancer.

Furthermore, in a lytic infection, HIV-1 protease cleaves host procaspase-8, generating a fragment called casp8p41. Casp8p41 can either bind to and activate mitochondrial pro-apoptotic protein BAK or bind to anti-apoptotic BCL-2 (in high concentrations) which sequesters Casp8p41 and prevents apoptosis (24). Venetoclax has been shown to reduce levels of HIV-1 DNA-integrated cells, reduce the reservoir size, and cause preferential killing of HIV-1 expressing cells *in vitro*. In HIV-1 infected cells, casp8p41 binds to BCL-2 and becomes unable to activate pro-apoptotic proteins causing cell survival (25). Venetoclax prevents casp8p41 from binding BCL-2 which allows casp8p41 to bind to BAK and induce apoptosis *via* the intrinsic cell-death pathway. Reactivation of the HIV-1 reservoir in the presence of high levels of BCL-2 prevented death of the reactivated cells, but the addition of venetoclax circumvented this problem. There was no significant impact on uninfected cell viability or proliferation (24, 26). The addition of venetoclax to *ex vivo* HIV-1 infected samples that were exposed to latency reversal agents (LRAs) and co-cultured with HIV-1-specific T cells enabled CD8+ T cell killing of HIV-1 infected cells. Venetoclax did not impair viability or functionality of CD8+ T cells (27). Taken together and considering BCL-2 is necessary for survival of proliferating latently infected cells, this suggests that venetoclax might block persistence and replenishment of the latent reservoir and boost the killing activity in the shock and kill approach to HIV-1 cure (26).

### BCL-2, SASP, and INK4a/ARF-perpetuated senescence

Senescence is a cellular process that induces stable growth arrest and limits the proliferation of aged cells. Senescence is induced by drivers of damage, and in turn, senescence drives some of the key markers of aging including stem cell exhaustion and chronic inflammation (28). There exists a wide range of triggers of senescence, including oxidative stress, mitochondrial dysfunction, replicative stress, cytokines, irradiation, and genotoxic agents. As a result of senescence, cells express a phenotype named senescence-associated secretory phenotype (SASP). SASP is comprised of pro-inflammatory cytokines, growth factors, and cytotoxic mediators. The expression of SASP can affect nearby cells *via* paracrine signaling and consequently convert them to senescence as well (29). There are some benefits to senescence, as the process helps facilitate embryonic morphogenesis and acts to suppress dysfunctional/aged cells. However, this comes at a cost, as senescent cells therefore accumulate in aged tissues, indicating that senescence could itself promote aging (28).

One of the primary factors driving senescence is damage to a cell’s telomeres. Telomeres are highly repetitive DNA structures located at the end of chromosomes that act to protect against chromosome degradation. However, with every successive round of cell cycle division, telomeres shorten (28). While cells have DNA repair mechanisms, the damage done to telomeres is hidden by the presence of specialized nucleoprotein complexes called shelterins. As a result of damaged telomeres, cells can no longer divide, thus driving senescence. This phenomenon is better known as telomere attrition (30).

It has been well-established that BCL-2 proteins play an integral role in the apoptotic pathway. However, it has also been revealed that BCL-2 may also play a role in cellular senescence. Specifically, BCL-2 has been seen to accelerate premature senescence in both human lung primary fibroblasts and human foreskin fibroblasts (31). The exact mechanism of how BCL-2 and senescence work in tandem is yet to be completely elucidated, but recent experiments have been able to yield results demonstrating the impact BCL-2 has on senescence.

Senescent cells upregulate BCL-2 and BCL-X_L_, both of which are anti-apoptotic proteins (32). When senescence in primary human fibroblasts were induced *via* DNA damage or replicative exhaustion and treated with tumor necrosis factor-α and cycloheximide to induce apoptotic pathway, senescent cells survived at a much higher rate compared to empty vector-transduced cells. Furthermore, levels of BCL-2 and BCL-X_L_ were detected to have increased in these senescent cells. When the cells were treated with a known small molecule inhibitor of anti-apoptotic proteins called ABT-737, high levels of cell death were seen in the senescent cells compared to the control (11). In human primary fibroblasts introduced with an activated *ras* allele, a similar phenomenon was discovered as well. The upregulation of BCL-2 resulting from the introduced *ras* allele increased the rate of appearance of senescence-associated β-galactosidase, a known marker of senescence. Compared to control cells with no *ras* allele, there was a higher percentage of senescence-associated β-galactosidase-positive cells in the cells with the *ras* allele (31).

These discoveries lend to the evidence that BCL-2 levels may decide if cells undergo senescence versus p53-dependent apoptosis. In the presence of high levels of BCL-2, the inhibition of apoptosis may commit cells towards a path of senescence instead (33). As a result, it is plausible to conclude that the expression of anti-apoptotic proteins such as BCL-2 and BCL-X_L_ contribute to the resistance of senescent cells towards programmed cell death.

### Senescence in cancer

Cellular senescence plays an important role as a tumor suppressor mechanism. Senescence has antiproliferative power and thus can restrict the progression of tumors (34). Like with aging, the inhibitor of cyclin-dependent kinase 4 alpha/alternate reading frame (*INK4a/ARF)* locus plays a significant role in cancer. Oncogenic mutations in cells activate the expression of the locus, thereby encoding tumor suppressing proteins such as p15^INK4b^ and p16^INK4a^. These proteins inhibit and inactivate cyclin-dependent kinase (CDK) 4/6, thus inducing cell cycle arrest and preventing continued division of cancer cells. The ARF protein inhibits mouse double minute homolog 2 (MDM2), a protein that usually inhibits the transcriptional activity of p53. As a result of the inhibition of MDM2, p53 is stabilized and leads to cell-cycle arrest, thus preventing further division of cancer cells as well (35).

Of note is that senescence in tumors is closely tied only to the premalignant states of tumorigenesis. In fact, senescent markers appear absent in tumors once they are in their malignant stages. These findings suggest that senescence acts as a barrier to tumor progression (36). Because of the interconnectedness between cancer pathways and senescence, potential cancer treatments have been targeted towards inducing senescence or blocking the cellular pathways that lead to the proliferation of cancer cells. For example, multiple chemotherapeutic drugs under investigation act to inflict severe DNA damage to cancerous cells, thus triggering cellular senescence. Further, some small molecule inhibitors are being developed to restore p53 signaling, as p53 function is lost in tumor cells; this could help control the rapid division of tumor cells.

### Relationship between senescence and aging

**Figure 3:**
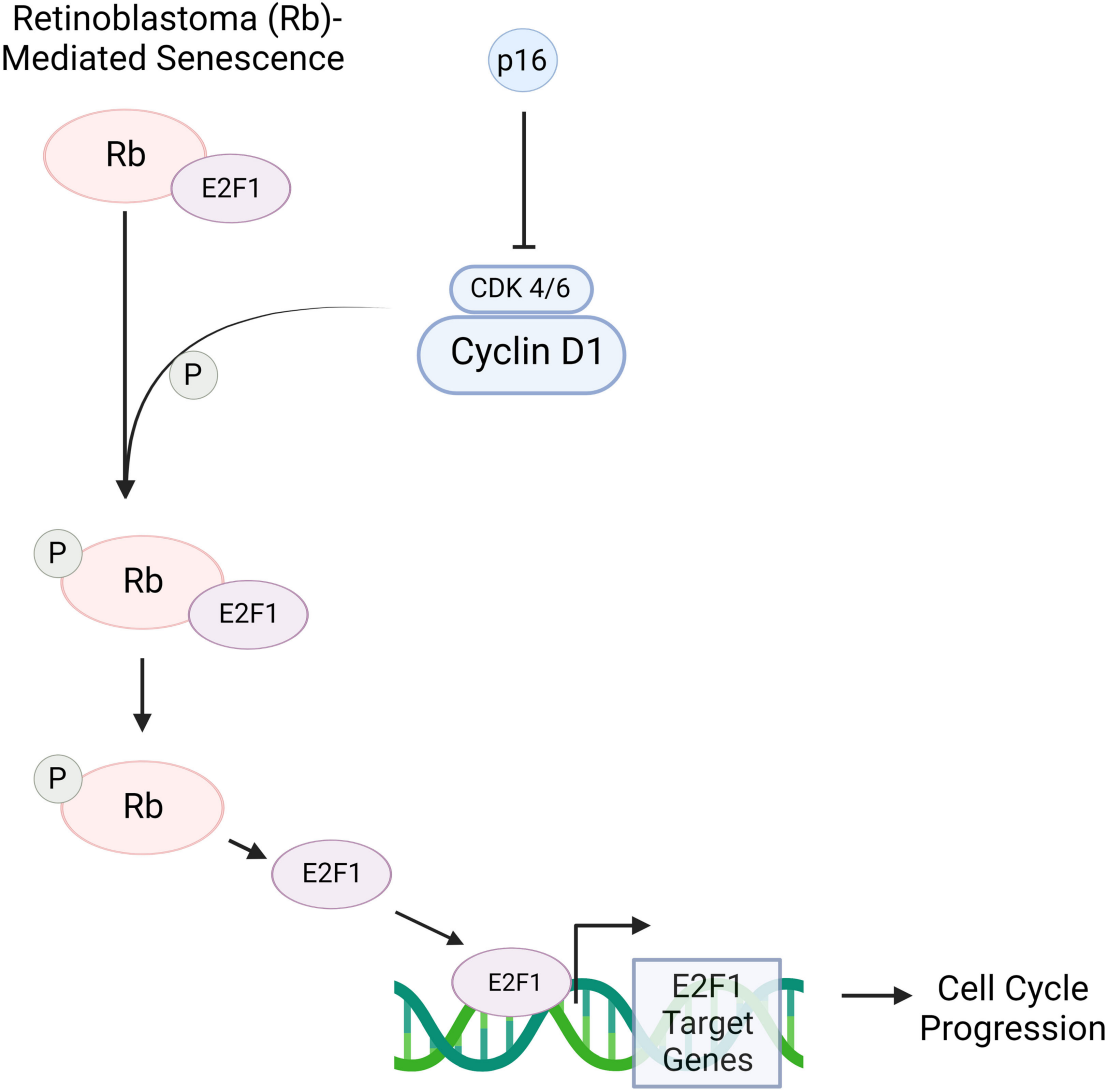
p16 is a protein that, when in increased concentrations, can induce senescence *via* the retinoblastoma (RB)- pathway. Rb is a tumor suppressor protein that plays a critical role in regulating the cell cycle. Cyclin D1 activates cyclin-dependent kinase 4/6 (CDK 4/6), allowing the kinase to phosphorylate the Rb-E2F1 complex. This complex consists of the Rb protein and E2F1 transcription factor. When phosphorylated, Rb is inactivated, allowing for the detachment of E2F1; consequently, E2F1 target genes are transcribed. These play a crucial role in the transition of a cell from the G1 to S phase. However, in the presence of p16, CDK 4/6’s ability to deactivate Rb through phosphorylation is inhibited. As a result, E2F1 remains bound to Rb, and therefore transcription of E2F1 target genes is prevented. This blocks cell cycle progression, thus causing a cell to move towards senescence. Created with BioRender.

The question of whether senescence and aging are related through causation is still under investigation, but there are components of senescence that do correlate with the process of aging. One of these components is the *INK4a/ARF* locus where three tumor suppressors reside: p16^INK4a^, ARF, and p15^INK4b^ (28). At a young age, the *INK4a/ARF* locus is expressed at low levels in most tissues but slowly becomes derepressed with continued aging (34). Specifically, the p16 protein is of particular interest as it is an important marker of senescence that has been observed to accumulate in aged tissues of mammals (37). P16-mediated senescence works *via* the retinoblastoma (Rb) pathway. p16 binds to CDK4/6 which thus inactivates the kinases and prevents the phosphorylation of the Rb protein. The lack of phosphorylation in Rb proteins causes them to remain associated with transcription factor E2F1 as an Rb-E2F1 complex. The restriction of the E2F1 transcription factor prevents transcription of E2F1 target genes which are otherwise crucial for the transition from the G1 to S phase in the cell cycle (**Figure 3**). Consequently, the increase in p16 concentration leads to cellular senescence by inhibiting the CDK pathway (38).

The aforementioned importance of p16 in senescence has been observed in progeroid mice. Usually, these mice show early onset of a few age-associated disorders such as cerebral gliosis and cataracts. In one experiment, the progeroid mice were engineered so cells expressing p16 would be induced to undergo apoptosis. In contrast to the unaltered mice, these mice displayed delayed onset in the age-associated disorders. In naturally aging INK-apoptosis through targeted activation of caspase (ATTAC) mice, the same alterations were made; delayed onset of the age-associated disorders was once again observed alongside an increase in lifespan. Altogether, this suggests that senescence plays a role in age-associated pathologies and that senescent cells may limit longevity (37).

SASP as a product of senescence can also contribute to the aging process. The paracrine transmission of the senescence response can contribute to chronic inflammation, or inflammaging. Elimination of senescent cells in aged organs has been seen to reduce levels of IL-6 and IL-1β, both of which are markers of chronic inflammation. Another process called immunosenescence is correlated with aging. The immune system in healthy individuals can usually clear senescent cells (28). However, with age, there is a decline in the production of adaptive immune cells (29). As a result, the clearance of senescent cells becomes compromised, leading to greater inflammation (28).

### BCL-2 interactions in SARS-CoV-2

A global pandemic has presented unique challenges in the scientific community. With the technology and means to quickly share data, facilitating collaborative problem-solving, we also see a lag in the peer-review process due to increased submission and importance of information at this time. This has led to some contention on severe acute respiratory syndrome coronavirus 2 (SARS-CoV-2) interactions with BCL-2 in the field. Ongoing research seeks to characterize SARS-CoV-2 but many predictions and comparisons are drawn based on existing knowledge of Middle East respiratory syndrome (MERS-CoV) and severe acute respiratory syndrome (SARS-CoV) and comparisons with related positive sense single stranded RNA viruses ((+)ssRNA).

It has been posited that because the mitochondria is targeted by related (+)ssRNA viruses, it may also be a target of SARS-CoV-2 (39). In observing the human interactome with MERS and SARS-CoV, it was predicted that BCL-2, MCL-1, and BCL-2A1 would be upregulated in SARS-CoV-2 infection (40). Rad9 is a human protein that has a BH3-like region and interacts with BCL-2 and BCL-X_L_ to induce apoptosis (41). Rad9A can purportedly be targeted by SARS-CoV-2 encoded miRNA that suppress the gene (preliminary data from Xingyin Liu’s group at Nanjing Medical University, China, doi: 10.48550/arXiv.2004.04874). This suggests that SARS-CoV-2 relies on BCL-2 for a productive infection. However, there is more evidence to support that SARS-CoV-2 likely downregulates BCL-2 instead.

SARS-CoV induces apoptosis *via* the intrinsic cell-death pathway and nasopharynx samples from SARS-CoV-2 patients have shown increased apoptosis and expression of caspase-3 (42–48). SARS-CoV infection induces caspase-dependent apoptosis which can be prevented by overexpression of BCL-2 or by caspase inhibitor (42, 48). SARS-CoV produces a 7a protein that induces apoptosis that can be blocked by overexpression of BCL-X_L_. 7a has been found to interact with BCL-X_L_, BCL-2, MCL-1, and BCL-A1 (46). SARS-CoV-2 shares the ORF7a gene with SARS-CoV, which makes it likely that SARS-CoV-2 7a protein has a similar interaction with these BCL-2 proteins (48). The SARS-CoV E protein causes apoptosis that can be prevented by BCL-X_L_ (44, 49). SARS-CoV-2 E protein has sequence similarity and highly conserved N-terminal regions with SARS-CoV, and so may share a similar function (50). SARS-CoV-2 infection in the lung increases expression of Noxa, an MCL-1 inhibitor, which increases apoptosis. Younger lung tissue has been found to be more primed for apoptosis because more cytochrome C is released during MOMP than in older lung tissue (51). In Vero cells, BCL-2 expression prevents apoptosis but does not affect viral infection, replication, or release which indicates that although SARS-CoV causes apoptosis, it is not necessary for viral dissemination (42). SARS-CoV-2 induces apoptosis *via* the extrinsic cell-death pathway through caspase-8 and caspase-9. Caspase-8 cleaves pro-apoptotic BID to tBID (truncated BID) which induces release of cytochrome C. This ultimately results in formation of the apoptosome and activation of caspase-9 which begins the caspase cascade leading to apoptosis (27).

Of note, Jak 1/2 inhibitor baricitinib was granted an emergency use authorization in 2020 for the treatment of severe COVID-19. Combination therapy of baricitinib and remdesivir was shown to reduce recovery time in patients receiving high-flow oxygen or ventilation compared to remdesivir alone. Serious adverse events were reduced from 21% with only remdesivir to 16% with baricitinib (52). The anti-inflammatory effect of baricitinib is especially useful in treating severe COVID-19 and COVID-19 pneumonia which progress due to severe inflammation in the lungs. As the Jak STAT pathway is an upstream regulator of BCL-2 expression, a potential connection between BCL-2 and severe COVID-19 may exist but needs further investigation.

### BCL-2 conservation among other viral species

With BCL-2 inhibitors proving effective against cancer and HIV-1, they could potentially be used as an “antiviral” against other viral species. Its potential efficiency would depend on how the pathogen(s) could lead to BCL-2 upregulation making the infected host-cell a long-lived reservoir.

### Upregulation of BCL-2

The following viruses upregulate BCL-2, which make them viable targets for a BCL-2 inhibitor. All gamma herpes viruses and adenoviruses encode viral BCL-2 protein homologs. Adenoviruses encode an anti-apoptotic BCL-2 protein homolog, E1B 19K, that does not have homology with BCL-2 proteins. E1B 19K interacts with BAX and BAK, so BH3 mimetics may also inhibit the homolog (53). Zaire ebolavirus induces lymphocyte apoptosis. Although lymphocytes are not infected, macrophages are and do not undergo apoptosis. BCL-2 upregulation has been documented by day 4 of infection. Because the lymphocyte apoptosis happens at later stages of infection, it is suspected to contribute to suppression of adaptive immunity, which is proposed to play a role in septic shock (54, 55). In human papillomavirus-induced squamous cell carcinoma of the cervix, BCL-2 is overexpressed (56). In picornavirus infection, IFN-β promoter stimulator 1 (IPS-1)-dependent IFN regulatory factor 3 (IRF-3) activation upregulates expression of BCL-2 (57). During infection, mitochondrion-anchored death signaling proteins can activate caspase-8 (1). BCL-2 proteins on the outside of the mitochondrial membrane oligomerize which forms pores that pro-apoptotic factors can access the cytoplasm through (1). Poxviruses encode an N1 anti-apoptotic protein that binds BH3 and prevents apoptosis (58). The crystallographic structure of N1 has revealed similarity to human BCL-2 (58). N1 binds to BH3 with high affinity (58). Similarly, to adenovirus E1B 19K, BH3 mimetics may be effective in inhibiting N1.

### Downregulation of BCL-2

The following viruses downregulate BCL-2 and so should not be treated with a BCL-2 inhibitor. Treatment plans should consider this aspect if co-infections are observed. Herpes-simplex virus 2 (HSV-2) and Hantaan virus both downregulate BCL-2 for a productive infection as they induce apoptosis in late-stage infection (59). Merkel cell polyomavirus (MCPyV) is associated with deregulated expression of BCL-2 (60). BCL-2 in Merkel cell carcinoma (MCC) patients indicates an earlier clinical stage and longer survival in patients. Downregulation of BCL-2 negative tumors results in local and systemic metastasis (61). Within the *Rhabdoviridae* family, BCL-2 controls release of cytochrome C and inhibits AIF translocation to the nucleus, preventing DNA damage (62). Within Madin-Darby canine kidney (MDCK) cells, BCL-2 expression has been shown to block orthomyxovirus-induced apoptosis and reduce production of infectious progeny (63). Adherence to seasonal flu vaccine may prevent this from being a problem in treatment plans.

## Immunomodulators affecting BCL-2 expression

There are both direct and indirect routes to target BCL-2. BH3 mimetics and BCL-2 anti-translational compounds are direct acting whereas compounds like Jak STAT inhibitors have downstream effects on BCL-2 expression. A comprehensive overview of all immunomodulators discussed in this section is provided in **Table 1**, including indirect and direct acting compounds.

**Table 1:** Report of drug class, cellular target, clinical trial status (phase I, II, III, approved, terminated), approval in children, oral bioavailability, route of clearance, compound half-life and dosing for Jak and BCL-2 inhibitors.

Compound	Class	Target	Clinical Status	Approved in Children	Orally Bioavailable	Clearance	Half-Life	Dosing
***Tofacitinib***	Jak STAT Inhibitor	Jak 3	FDA approved	No	Yes	Predominantly hepatic	3 Hours	5-10 mg
***Ruxolitinib***	Jak STAT Inhibitor	Jak 1/2	FDA approved	No	Yes	Predominantly renal	3 Hours	5-25 mg
***Baricitinib***	Jak STAT Inhibitor	Jak 1/2	FDA approved	Yes (2-17)	Yes	Renal	12 Hours	2-4 mg
***Venetoclax***	BH3 Mimetic	BCL-2 Specific	FDA approved	No	Yes	Hepatic	26 Hours	10-100 mg
***Oblimersen***	Phosphorothioate antisense oligonucleotide	BCL-2 mRNA translation	Multiple phase III/ ceased manufacture	No	No	Minimally renal / Predominant not reported	2.5 Hours	3 mg/kg/day
***ABT-737***	BH3 Mimetic	Pan-BCL-2 inhibitor (BCL-2, BCL-XL, BCL-W)	Pre-clinical	No	No	N/A	N/A	1 µM (sub-cytotoxic)
***Navitoclax***	BH3 Mimetic/ Senolytic	Pan-BCL-2 inhibitor (BCL-2, BCL-XL, BCL-W)	Multiple phase II/ recruiting multiple phase III	Yes (Birth-17)	No	Not renal/ Predominant not reported	17 Hours	150 mg
***Obatoclax***	BH3 Mimetic	MCL-1	Multiple phase II/ ceased manufacture	No	No	N/A	7-14 Hours	MTD (28 mg/m2 over 3 hours every 3 weeks)
***AT-101***	BH3 Mimetic	BCL-2, BCL-XL	Multiple phase II/ ceased manufacture	No	Yes	N/A	N/A	N/A

Some data are undefined for these compounds and are marked as not available (N/A).

### BCL-2 inhibitors

The first BCL-2 inhibitor that progressed to clinical trials was an antisense oligodeoxynucleotide that targeted BCL-2 mRNA. This inhibitor was used with chemotherapy to treat chronic lymphocytic leukemia (CLL) (64). BH3 mimetics are a largely researched class of BCL-2 inhibitor. Within the BCL-2 protein family, some proteins have a BH1, BH2, and BH3 domain while some only have the BH3 domain, called BH3-only proteins (65). This homology makes the BH3 domain an ideal target. The BH3 domain on the BCL-2 proteins is where the BH3 peptide binds and thus becomes unable to activate pro-apoptotic proteins. The BH3 mimetics can sequester the BCL-2 anti-apoptotic proteins in high concentrations which allows for pro-apoptotic protein activation and subsequent cell death.

### Oblimersen

Oblimersen was the first developed BCL-2 inhibitor. Oblimersen is a phosphorothioate antisense oligonucleotide that inhibits BCL-2 mRNA translation. In pre-clinical studies, the dosage was described as 3 mg/kg/day with a half-life of 2.5 hours and with minimal renal clearance (66). There are several completed phase III clinical trials for oblimersen for melanoma, adult myeloid leukemia, multiple myeloma and plasma cell neoplasm and leukemia. However, oblimersen did not show efficacy in increasing patient survival in phase III trials studying lymphoma, which lead to the FDA rejecting the drug twice and subsequent withdraw of the new drug application (NDA) for oblimersen by the pharmaceutical company Genta in 2004 (67, 68).

### ABT-737

ABT-737 is a BH3 mimetic pan-BCL-2 inhibitor, targeting BCL-2, BCL-X_L_, and BCL-W (69). However, if MCL-1 is overexpressed in target cells, ABT-737 will not induce apoptosis (70). MCL-1 can also sequester BAK, and so this drug may be useful in combination with others (71, 72). Pre-clinical data defines a sub-cytotoxic dosage of 1 µM (73). ABT-737 was found to eliminate senescent cells in mice (11). A pre-clinical ex vivo study of ABT-737 with ovarian cancer (ClinicalTrials.gov Identifier: NCT01440504) is the only clinical trial data available for this compound. Studies with this drug lead to the development of navitoclax (74).

### Navitoclax

Navitoclax, previously ABT-263, is an orally bioavailable pan-BCL-2 inhibitor that binds to BCL-2, BCL-X_L_, and BCL-W. Navitoclax is a BH3 mimetic. Pre-clinical data describes the dosage as 150 mg with a half-life of 17 hours and clearance not occurring renally (75, 76). The European Medicines Agency (EMA) granted a waiver for use of this drug in pediatrics, ages birth to 17 years, to treat myelofibrosis in 2018 (77). Navitoclax is also considered a senolytic. Other senolytics include dasatinib, quercetin, and fisetin although these are not BCL-2 inhibitors. Interestingly, fisetin is a senolytic flavonoid which has BCL-2 inhibiting activity and was found to reduce mortality in β-coronavirus infected mice (78). This finding has led to an imminent FDA-approved clinical trial (79). There are several clinical trials with Navitoclax currently recruiting: phase II with ruxolitinib for both myelofibrosis and relapsed/refractory myelofibrosis, phase I for myeloproliferative neoplasm, phase III for a tolerability and efficacy combination study with ruxolitinib for myelofibrosis, phase I/II with vistusertib for relapsed small cell lung cancer, phase I/II with trametinib for metastatic, refractory, and unresectable malignant solid neoplasms, and a phase I/II with dabrafenib and trametinib for malignant solid neoplasm, metastatic melanoma, state II (A,B,C) and IV cutaneous melanoma AJCC v7, and unresectable melanoma.

### Obatoclax

Obatoclax mesylate, a mesylate salt of obatoclax, is a BH3 mimetic that binds to MCL-1 and has been studied in combination with other drugs against cancer in clinical trials. Pre-clinical data describes the maximum tolerated dose as 28 mg/m (2) over 3 hours every 3 weeks and a half-life of 7-14 hours (80). Several phase II clinical trials have been completed for acute myeloid lymphoma, in combination with rituximab for follicular lymphoma, Hodgkin’s lymphoma, myelodysplastic syndromes, myelofibrosis, in combination with caroplatin/etoposide for extensive stage small cell lung cancer, in combination with bortezomib for mantle cell lymphoma, in combination with docetaxel for lung cancer, and in combination with topotecan hydrochloride for recurrent small cell lung cancer. Developers terminated clinical trials in 2012 and discontinued the drug with no updates since (67).

### AT-101

AT-101, also referred to as gossypol acetic acid, is an orally bioavailable BH3 mimetic. AT-101 binds to BCL-2 and BCL-X_L_. It has undergone phase II clinical trials and it currently being investigated for efficacy against solid tumors (67). AT-101 has been investigated in multiple completed phase II clinical trials for chronic lymphocytic leukemia, in combination with prednisone and docetaxel for hormone refractory prostate cancer, in combination with docetaxel for non-small cell lung cancer, for recurrent and stage III/IV adrenocortical carcinoma, for relapsed/refractory B-cell malignancies, in combination with rituximab for follicular lymphoma, for progressive or recurrent glioblastoma multiforme, for extensive stage and recurrent small cell lung cancer, in combination with bicalutamide for adenocarcinoma of the prostate and stage IV prostate cancer, for prostate cancer, in combination with topotecan for small cell lung cancer, and for hormone refractory prostate cancer. Manufacturing of AT-101 was ceased in 2009.

### Venetoclax

Venetoclax, previously ABT-199, is a BH3 mimetic that is BCL-2-specific. Venetoclax was the first BCL-2 inhibitor to be FDA-approved (81). Venetoclax is an approved treatment for acute myeloid lymphoma (MCL), CLL, and small lymphocytic leukemia (SLL) (82, 83). Venetoclax has a dosage of 10-100 mg with a half-life of 26 hours and is orally bioavailable. Venetoclax is contraindicated for strong inhibitors of CYP3A (84). Recently, venetoclax has been experimentally shown to decrease the size of the HIV-1 reservoir (26). Venetoclax has demonstrated ability to penetrate into the CNS compartment, however Venetoclax is highly bound to plasma proteins (> 99%), which may limit its tissue penetration and subsequent free active drug concentrations across two-compartment pharmacokinetic models (84, 85). To date, these data are not defined, however studies are warranted across one and two compartment pharmacokinetic modeling efforts to better define potential for Venetoclax to decay the reservoir systemically. While tissue penetrability would be a desirable trait considering myeloid viral reservoirs and tissular tumor environments, tissue distribution of Venetoclax has not been formally documented but likely has limited tissue distribution to secondary lymphoid organs as it is highly bound to plasma protein (84).

## Jak STAT Inhibitors

### Tofacitinib

Tofacitinib is a Jak 1/2/3 inhibitor that is FDA approved for the treatment of rheumatoid arthritis and is approved for chronic long-term use. The dosage for tofacitinib is 5-10 mg with a half-life of 3 hours and oral bioavailability of 74%. 70% of clearance occurs hepatically with the remaining 30% being renal. Tofacitinib can cause lymphocytosis, neutropenia, and anemia which diminishes its desirability as a therapy for infection of immune cells. There is a black box warning for observed infections leading to hospitalization or death, lymphoma and other malignancies, and an increased rate of Epstein-Barr virus-associated post-transplant lymphoproliferative disorder in renal transplant patients treated concomitantly with immunosuppressive medication [Venetoclax package insert].

### Ruxolitinib

Ruxolitinib is a Jak 1/2 inhibitor that is FDA approved for treatment of myelofibrosis and is approved for chronic long-term use. At a dosage of 5-25 mg, ruxolitinib has a half-life of 3 hours and is 95% orally bioavailable. Renal clearance is the predominant route of excretion at 74% with hepatic clearance at 22% [Jakafi package insert]. A recent human clinical trial with PWH treated for 5 weeks with ruxolitinib demonstrated reduced biomarkers of inflammation, immune dysregulation, T-cell activation, cellular lifespan, and gut microbial translocation while increasing IL-7R expression which is inversely related to homeostatic proliferation (7).

In studies with HIV-1, ruxolitinib and tofacitinib inhibit IL-2, IL-7, and IL-15-induced STAT5 phosphorylation and BCL-2 expression. IL-2, IL-7, and IL-15 which all signal through Jak STAT, have integral roles in HIV-1 pathogenesis. IL-2 increases viral reactivation. IL-7 and IL-15 drive homeostatic proliferation and promote survival and proliferation of latently infected cells. pSTAT5 strongly correlates with increased integrated viral DNA levels, directly contributing to HIV-1 persistence (3). Ruxolitinib and tofacitinib have been shown to inhibit virus replication in lymphocytes stimulated (phytohemagglutinin (PHA) + IL-2) for reactivation and inhibit reactivation in central memory lymphocytes (9). These studies highlight the link between the Jak STAT pathway and HIV-1 reservoir expansion and maintenance.

### Baricitinib

Baricitinib is also a Jak 1/2 inhibitor with FDA approval for the treatment of rheumatoid arthritis and an emergency use authorization for COVID-19 (86). Of the three Jak STAT inhibitors discussed herein, baricitinib possess the most favorable safety profile. Baricitinib is administered quaque die orally at 2-4 mg and is blood-brain-barrier penetrant. Baricitinib is 80% orally bioavailable, reaching peak plasma concentrations within an hour of administration, and with an elimination half-life of 12 hours. Baricitinib is approved for chronic long-term use, including in children aged 2-17. Baricitinib is cleared renally, lowering chances of hepatic drug-drug interactions. There is a black box warning for observed infections leading to hospitalization and death, lymphoma and other malignancies, and thrombosis [Olumiant package insert]. More than 50 human trials of baricitinib for inflammatory disorders are currently underway. In the context of HIV-1 infection, basal level inflammation associated with the HIV-1 reservoir leads to long-term complications such as HIV-associated neurocognitive dysfunction (HAND), HIV-associated dementia (HAD), and cardiovascular disease (CVD) (87–89). To date, the tissue penetration profile of Jak 1/2 or BCL-2 inhibitors is not fully elucidated in humans across all tissues and organs. Baricitinib is not fully plasma protein-bound, highlighting its potential to penetrate tissues as an unbound, free agent. We previously proposed baricitinib as a potential therapy to target immune dysregulation resulting from HIV infection in both the lymphocytic and myeloid compartments (90). A recently NIH funded Phase 2a trial with baricitinib to evaluate the ability of this agent to reduce the HIV-1 reservoir in the CNS compartment is now underway (NCT05452564), underscoring potential for baricitinib to not only penetrate across compartments such as, but not limited to the CNS. Further, the data from this forthcoming trial will provide additional pharmacokinetic and pharmacodynamic properties of baricitinib towards reduction of the reservoir not only peripherally but across compartments; these data will be sentinel towards understanding how baricitinib can potentially be utilized in cure-based regimens for PWH.

Overall, baricitinib presents ideal qualities for concomitant use with ART including: a long half-life, no contraindications with ART or other antiretroviral therapies, renal clearance to avoid potential drug-drug interactions with hepatically cleared ART, orally bioavailable for ease of administration, and blood-brain-barrier penetrant to target infected cells in the central nervous system (CNS), a notorious sanctuary site in HIV-1 infection.

## Conclusion

Several viruses and cancers have evolved to overcome the efficient immunity to promote its own survival. BCL-2 inhibitors have the potential to hinder viremia in viral infections where BCL-2 anti-apoptotic proteins are upregulated, but when these anti-apoptotic proteins are downregulated a BCL-2 inhibitor could aid infection and have drastic effects on a patient’s health. A BCL-2 inhibitor alone may not be effective as an anti-viral, but in combination therapy could be beneficial. Venetoclax, an existing FDA-approved BCL-2 inhibitor, could be repurposed as an antiviral against viruses that upregulate BCL-2 anti-apoptotic proteins in infection. Combination therapy of multiple BCL-2 inhibitors or development of more broadly inhibiting BH3 mimetics would likely be more effective in targeting senescent cells and potentially infected cells and tumors. SARS-CoV-2 is projected to downregulate BCL-2 anti-apoptotic proteins, so a BCL-2 inhibitor could exacerbate cellular damage during infection. Treatment plans should consider co-infections and viral infections in individuals with cancer before incorporating BCL-2 inhibitors so as not to further impair a patient’s health. Overall, BCL-2 proteins present a target that could be exploited in some viral infections and BCL-2 inhibitors could further expand to be used as antivirals.

## Author contributions

MDR (majority) and CS performed literature research, writing, and figure preparation. SH contributed to literature search and writing. MDR prepared the manuscript. SPR, DK, and CG contributed topic conception and evolution as well as provided critical revision. All authors have reviewed the final version of materials and approved for submission.

## Funding

5T32AI106699-08 (NIAID) support of graduate training for MR 5UM1AI164562-02 (NIAID, NHLBI, NIDA, NIDDK, NINDS) support of DK 1R01MH128158-01A1 (NIMH) and 1R01HL166004-01 (NHLBI) support of CG 1UM1AI164561-01 (NIAID) support of MR and CG.

## Acknowledgements

We would like to thank Emory University’s Microbiology and Molecular Genetics doctoral program, Pathology and Laboratory Medicine department, as well as the Emory Vaccine Center at Yerkes National Primate Research Center for support of this work. We would also like to thank William Shafer for guidance through the writing process and commitment to teaching.

## Conflict of interest

Author CG and Emory University receive royalties from Eli Lilly and Company for the sales of Baricitinib for the indication of COVID-19.

The remaining authors declare that the research was conducted in the absence of any commercial or financial relationships that could be construed as a potential conflict of interest.

## Publisher’s note

All claims expressed in this article are solely those of the authors and do not necessarily represent those of their affiliated organizations, or those of the publisher, the editors and the reviewers. Any product that may be evaluated in this article, or claim that may be made by its manufacturer, is not guaranteed or endorsed by the publisher.
